# *Streptococcus mitis/oralis* Corneal Ulcer in a Patient with Severe Rheumatoid Arthritis: An Unusual Route

**DOI:** 10.1155/2022/5245620

**Published:** 2022-08-16

**Authors:** Mustafa Jaffry, Mark Lister

**Affiliations:** ^1^Rutgers New Jersey Medical School, Newark NJ, USA; ^2^New York Medical College, Valhalla, New York, USA

## Abstract

**Purpose:**

To report a case of Streptococcus mitis oralis (*S. mitis/oralis*) corneal ulcer in a patient with a possible preventable cause.

**Methods:**

Cultures were obtained from a 64-year-old woman with a 3.5 mm × 5 mm corneal ulcer with hypopyon in the left eye.

**Results:**

Culture reports demonstrated the growth of S*. mitis/oralis*, a commensal organism of the oral flora. Fortified vancomycin 5% eye drops were started, and the patient was counseled on the rarity of the bacteria as an etiology of corneal ulcers. On the return visit, the patient, who works in a doctor's office, volunteered information that the preservative free artificial tear vials that she used were difficult to open because of her hand deformity due to rheumatoid arthritis; thus, she had to bite the vials open.

**Conclusions:**

*S. mitis/oralis* is an organism commonly found in the mouth but is rarely found in the eye. We report a unique case of an immunocompromised patient with rheumatoid arthritis, severe dry eye, and a history of multiple episodes of keratolysis, who developed a corneal ulcer from a rare pathogen, with a plausible and preventable route of infection.

## 1. Introduction

Streptococcus mitis (*S. mitis*) is an alpha-hemolytic gram-positive coccus and belongs to the group of viridans streptococci. Streptococcus oralis (*S. oralis*) belongs to the mitis group of bacteria, and direct speciation is often difficult in clinical settings. Usually isolated as part of the oral flora of the mouth, *S. mitis/oralis* is known to have a low virulence but can be opportunistic and cause severe infections such as endocarditis, meningitis, and bacteremia [1]. Corneal ulcers due to *S. mitis/oralis* have only rarely been described in the literature, limited to a few case reports in a variety of contexts. Herein, we report a unique case of corneal ulcer caused by *S. mitis/oralis* in an immunocompromised patient with severe rheumatoid arthritis (RA).

## 2. Case Report

A 64-year-old woman with a long history of RA and severe ulnar deviation of both hands presented with a central corneal ulcer in a previous corneal transplant of the left eye. She had a long history of multiple episodes of keratolysis in both eyes secondary to severe dry eye and RA. Throughout the course of her disease she had been on oral prednisone, methotrexate, hydroxychloroquine, intravenous immunoglobulin, and multiple immunosuppressants. Punctal plugs were placed in all 4 lids. The patient had a history of corneal melt in right eye initially, which resolved with oral steroids but resulted in significant scarring. Subsequently, she developed keratolysis in the left eye, which resulted in perforation and required a corneal patch graft that had difficulty healing. The patch graft continued to struggle with reepithelialization, even after multiple amniotic membranes were placed. The patient then developed a suspected fungal infection in the left cornea and underwent a full-thickness corneal transplant. Fungal cultures were not performed. Two months later, after natamycin therapy, there were no signs of residual fungus.

At this time, the patient underwent retinal surgery for retinal detachment in the left eye. Two weeks later, the patient developed a corneal ulcer with hypopyon in the same eye. Fortified vancomycin 5% was started every half hour. Cultures were taken and demonstrated moderate growth of *Streptococcus mitis/oralis*. Susceptibility testing was not performed, as is usual, because this is a commensal organism of the oral flora. The patient had no recent dental procedures and was counseled and alerted that this is an unusual bacterium that infects the eye and is commonly found in the mouth. Two days later, the hypopyon had decreased, and the ulcer was smaller. On follow up, 2 days after this, the hypopyon had decreased considerably and the patient, who works in a doctor's office, volunteered information on how she thought she may have gotten this infection. The patient reported “Since my hands are so deformed from the arthritis, I am unable to open the vials of preservative-free artificial tears. So, I have been biting off the tops of the vials, so I could insert the tears.” We concurred that oral bacteria from her mouth could have contaminated the vials before drop application. On follow-up, the corneal ulcer had quickly resolved.

## 3. Discussion

Corneal ulcers due to *S. mitis/oralis* represent a diagnostic challenge because of their rarity and have infrequently been described in the literature. In addition to the mouth, *S. mitis/oralis* can be found on the skin, gastrointestinal tract, and female genital tract. Rahman et al. studied microbial contamination in preservative-free eye drops in multidose containers and found significant growth in 8 out of 95 bottles tested. It was observed that *Staph aureus* was the most common contaminant [2]. Preservative-free artificial tears, which this patient used, came in single-use vials, an advantage of which, reduces the risk of contamination with multidose containers. Nevertheless, this patient's method of opening the bottle likely served as a route of infection. Although this patient had prior retinal detachment surgery, which could be another possible route for *S. mitis/oralis*, those complications usually lead to endophthalmitis and not corneal infection [3]. Our patient, prior to the corneal ulcer caused by *S. mitis/oralis*, also had suspected fungal keratitis, highlighting the vulnerability of the patient's cornea and the likely presence of epithelial defects. Although corneal ulcers caused by *S. mitis/oralis* are rare, some cases have been reported in the literature. It is conceivable that the immunocompromised state, along with the multiple ocular morbidities present in the patient, allowed for normally low virulence bacteria to cause a devastating infection. Khan et al. described a case of *S. mitis/oralis* causing a corneal ulcer after transplantation and reported that this was likely due to a reduction in local immunity after the surgery, opening the way for an opportunistic infection [4]. In another report, Hsiao et al. described a patient with recurrent corneal ulcers and a corneal culture that grew *S. mitis/oralis*. Topical vancomycin and ceftriaxone were administered, and the ulceration gradually resolved after amniotic membrane placement [5].

Rheumatoid corneal melt or keratolysis is the most severe complication of peripheral ulcerative keratitis (PUK), a rare form of ocular inflammation associated with rheumatoid arthritis (RA). PUK that results in perforation, caused by RA has been reported to have an incidence of 0.234 per million per year [6]. Corneal melt refers to the severe corneal stromal thinning that leads to perforation and is associated with abrupt loss of vision. PUK is described as inflammation of the limbal area of the cornea and is characterized by collagen destruction, infiltration, and vascular changes [7]. The current understanding of the pathogenesis of corneal melt as a consequence of PUK is unknown, but is thought to be autoimmune mediated inflammation, which results in unstable corneal epithelia, leading to vulnerability and a greater risk of infection as evidenced in this patient. The patient's severe RA was also demonstrated by the deformation of her hands bilaterally with ulnar deviation. This led to a difficulty in opening vials for preservative-free artificial tears, potentially contaminating the tear solution and infecting the eye.

In our case, fortified vancomycin was sufficient to resolve the infection with *S. mitis/oralis*, which agrees with the prior literature that has treated this pathogen [8]. Clinicians should be aware that biting bottles may be a method by which some patients cope with their disability. In this case report, we describe a patient with a corneal ulcer caused by *S. mitis* that was successfully resolved with antibiotics. We stress the importance of educating patients on sterile methods for applying eye drops, to avoid contamination. We also emphasize the importance of counseling patients and following up on how they may use their eye drops, especially in the elderly and those with physical difficulty. We advocate for the development of vials that are easier to use for patients with RA or other deforming arthropathies. This unique case highlights the importance of having accessible tear vials available to patients who cannot open them in the conventional way. With careful observation, close follow-up and open communication, immunocompromised patients at risk of opportunistic ocular infections can be protected. Anecdotally, cases of ocular infections caused by *S. mitis/oralis* have been increasing. A possible hypothesis for this could be widespread mask use, with contaminating fomites of oral bacteria from an individual's breath moving up from the mask and infecting the eye, which has been recently suggested [9]. However, no studies have yet been done to explore this.
